# Ethyl 1-[3-(1*H*-imidazol-1-yl)prop­yl]-2-(4-chloro­phen­yl)-1*H*-benzo[*d*]imidazole-5-carboxyl­ate dihydrate

**DOI:** 10.1107/S1600536811033654

**Published:** 2011-08-27

**Authors:** Yeong Keng Yoon, Mohamed Ashraf Ali, Ang Chee Wei, Ching Kheng Quah, Hoong-Kun Fun

**Affiliations:** a Institute for Research in Molecular Medicine, Universiti Sains Malaysia, 11800 USM, Penang, Malaysia; b X-ray Crystallography Unit, School of Physics, Universiti Sains Malaysia, 11800 USM, Penang, Malaysia

## Abstract

In the title compound, C_22_H_21_ClN_4_O_2_·2H_2_O, the almost-planar benzimidazole ring system [maximum deviation 0.014 (1) Å] is inclined at angles of 36.32 (5) and 74.75 (7)° with respect to the phenyl and imidazole rings, respectively. In the crystal structure, the water mol­ecules are linked to the organic mol­ecules to form a three-dimensional network via O—H⋯N and O—H⋯O hydrogen bonds. The packing is further consolidated by a pair of bifurcated C—H⋯O bonds, generating *R* ^1^_2_(6) loops. C—H⋯π inter­actions are also observed.

## Related literature

For related structures and background to benzimidazoles, see: Eltayeb *et al.* (2009 ▶, 2011 ▶). For standard bond-length data, see: Allen *et al.* (1987 ▶). For hydrogen-bond motifs, see: Bernstein *et al.* (1995 ▶). For the stability of the temperature controller used for the data collection, see: Cosier & Glazer (1986 ▶).
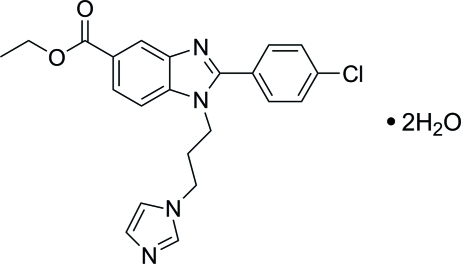


## Experimental

### 

#### Crystal data

C_22_H_21_ClN_4_O_2_·2H_2_O
*M* *_r_* = 444.91
Monoclinic, 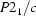

*a* = 9.0611 (1) Å
*b* = 13.8393 (2) Å
*c* = 18.0470 (3) Å
β = 92.386 (1)°
*V* = 2261.12 (6) Å^3^
*Z* = 4
Mo *K*α radiation
μ = 0.20 mm^−1^
*T* = 100 K
0.40 × 0.30 × 0.27 mm

#### Data collection

Bruker SMART APEX II CCD diffractometer
Absorption correction: multi-scan (*SADABS*; Bruker, 2009 ▶) *T*_min_ = 0.922, *T*_max_ = 0.947
31604 measured reflections
8235 independent reflections
6221 reflections with *I* > 2σ(*I*)
*R*_int_ = 0.030

#### Refinement

*R*[*F* ^2^ > 2σ(*F* ^2^)] = 0.048
*wR*(*F* ^2^) = 0.136
*S* = 1.05
8235 reflections
296 parameters
H atoms treated by a mixture of independent and constrained refinement
Δρ_max_ = 0.54 e Å^−3^
Δρ_min_ = −0.40 e Å^−3^

### 

Data collection: *APEX2* (Bruker, 2009 ▶); cell refinement: *SAINT* (Bruker, 2009 ▶); data reduction: *SAINT*; program(s) used to solve structure: *SHELXTL* (Sheldrick, 2008 ▶); program(s) used to refine structure: *SHELXTL*; molecular graphics: *SHELXTL*; software used to prepare material for publication: *SHELXTL* and *PLATON* (Spek, 2009 ▶).

## Supplementary Material

Crystal structure: contains datablock(s) global, I. DOI: 10.1107/S1600536811033654/hb6369sup1.cif

Structure factors: contains datablock(s) I. DOI: 10.1107/S1600536811033654/hb6369Isup2.hkl

Supplementary material file. DOI: 10.1107/S1600536811033654/hb6369Isup3.cml

Additional supplementary materials:  crystallographic information; 3D view; checkCIF report

## Figures and Tables

**Table 1:** Hydrogen-bond geometry (Å, °) *Cg*1 is the centroid of the C1–C6 phenyl ring.

*D*—H⋯*A*	*D*—H	H⋯*A*	*D*⋯*A*	*D*—H⋯*A*
O1*W*—H1*W*1⋯N1^i^	0.94 (3)	1.96 (3)	2.8802 (14)	164 (2)
O1*W*—H2*W*1⋯O2*W*^ii^	0.91 (2)	1.83 (2)	2.7284 (18)	169 (2)
O2*W*—H1*W*2⋯N4^iii^	0.849 (19)	1.978 (19)	2.8147 (19)	169 (2)
O2*W*—H2*W*2⋯O2^i^	0.87 (2)	1.98 (2)	2.8460 (17)	172 (2)
C17—H17*B*⋯O1*W*^iv^	0.99	2.49	3.3785 (16)	149
C19—H19*B*⋯O1*W*^iv^	0.99	2.51	3.3799 (19)	147
C10—H10*A*⋯*Cg*1^v^	0.95	2.86	3.4875 (14)	125

Symmetry codes: (i) 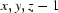; (ii) 
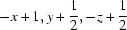
; (iii) 
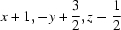
; (iv) 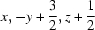; (v) 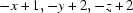.

**Fig. 1.**
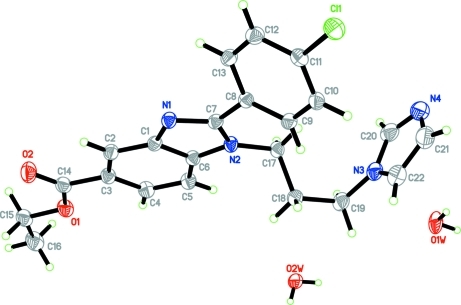
The molecular structure of the title compound showing 50% probability displacement ellipsoids for non-H atoms.

**Fig. 2.**
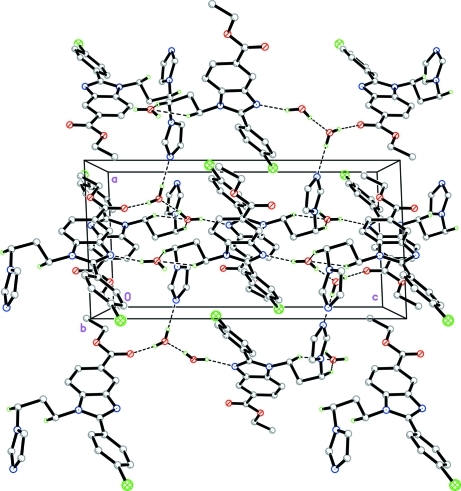
The crystal structure of the title compound, viewed along the b axis. H atoms not involved in hydrogen bonds (dashed lines) have been omitted for clarity.

## supplementary crystallographic information

### Comment

As part of our ongoing structural studies of benzimidazole derivatives (Eltayeb *et al.*, 2011), we now report the structure of the title compound, (I), which crystallised as a dihydrate.

In the title molecule, Fig. 1, the benzimidazole ring system (N1/N2/C1–C7, maximum deviation of 0.014 (1) Å at atom C4) is inclined at angles of 36.32 (5) and 74.75 (7)° with respect to the phenyl (C8–C13) and imidazole (N3/N4/C20–C22 ) rings. Bond lengths (Allen *et al.*, 1987) and angles are within normal ranges and are comparable to those in related structures (Eltayeb *et al.*, 2009, 2011).

In the crystal, water molecules are linked to main molecules to form a three-dimensional network (Fig. 2) by O1W—H1W1···N1, O1W—H2W1···O2W, O2W—H1W2···N4 and O2W—H2W2···O2 hydrogen bonds (Table 1). The crystal packing is further consolidated by bifurcated C17—H17B···O1W and C19—H19B···O1W acceptor bonds, generating *R*^1^_2_(6) ring motifs (Bernstein *et al.*, 1995). The crystal structure is also stabilized by C10—H10A···Cg1 (Table 1) interactions, where Cg1 is the centroid of the C1–C6 phenyl ring.

### Experimental

Ethyl 4-(3-(1*H*-imidazol-1-yl)propylamino)-3-aminobenzoate (0.84 mmol) and sodium metabisulfite adduct of chlorobenzaldehyde (1.68 mmol) were dissolved in DMF. The reaction mixture was refluxed at 403 K for 2 h. After completion, the reaction mixture was diluted in ethyl acetate (20 ml) and washed with water (20 ml). The organic layer was collected, dried over Na_2_SO_4_ and then evaporated in vacuo to yield the product. The product was recrystallised from ethyl acetate to yield bronze blocks of (I).

### Refinement

O-bound H atoms were located from the difference Fourier map and refined freely [O—H = 0.85 (2)–0.94 (3) Å]. The remaining H atoms were positioned geometrically and refined using a riding model with C—H = 0.95-0.99 Å and *U*_iso_(H) = 1.2 *U*_eq_(C). A rotating-group model was applied for the methyl group.

### Crystal data

**Table:**

C_22_H_21_ClN_4_O_2_·2H_2_O	*F*(000) = 936
*M**_r_* = 444.91	*D*_x_ = 1.307 Mg m^−^^3^
Monoclinic, *P*2_1_/*c*	Mo *K*α radiation, λ = 0.71073 Å
Hall symbol: -P 2ybc	Cell parameters from 9994 reflections
*a* = 9.0611 (1) Å	θ = 2.3–32.3°
*b* = 13.8393 (2) Å	µ = 0.20 mm^−^^1^
*c* = 18.0470 (3) Å	*T* = 100 K
β = 92.386 (1)°	Block, bronze
*V* = 2261.12 (6) Å^3^	0.40 × 0.30 × 0.27 mm
*Z* = 4	

### Data collection

**Table:**

Bruker SMART APEX II CCD diffractometer	8235 independent reflections
Radiation source: fine-focus sealed tube	6221 reflections with *I* > 2σ(*I*)
graphite	*R*_int_ = 0.030
φ and ω scans	θ_max_ = 32.7°, θ_min_ = 2.3°
Absorption correction: multi-scan (*SADABS*; Bruker, 2009)	*h* = −13→12
*T*_min_ = 0.922, *T*_max_ = 0.947	*k* = −15→20
31604 measured reflections	*l* = −27→27

### Refinement

**Table:**

Refinement on *F*^2^	Primary atom site location: structure-invariant direct methods
Least-squares matrix: full	Secondary atom site location: difference Fourier map
*R*[*F*^2^ > 2σ(*F*^2^)] = 0.048	Hydrogen site location: inferred from neighbouring sites
*wR*(*F*^2^) = 0.136	H atoms treated by a mixture of independent and constrained refinement
*S* = 1.05	*w* = 1/[σ^2^(*F*_o_^2^) + (0.067*P*)^2^ + 0.584*P*] where *P* = (*F*_o_^2^ + 2*F*_c_^2^)/3
8235 reflections	(Δ/σ)_max_ = 0.001
296 parameters	Δρ_max_ = 0.54 e Å^−^^3^
0 restraints	Δρ_min_ = −0.40 e Å^−^^3^

### Special details

**Table:**

Experimental. The crystal was placed in the cold stream of an Oxford Cryosystems Cobra open-flow nitrogen cryostat (Cosier & Glazer, 1986) operating at 100.0 (1) K.
Geometry. All esds (except the esd in the dihedral angle between two l.s. planes) are estimated using the full covariance matrix. The cell esds are taken into account individually in the estimation of esds in distances, angles and torsion angles; correlations between esds in cell parameters are only used when they are defined by crystal symmetry. An approximate (isotropic) treatment of cell esds is used for estimating esds involving l.s. planes.
Refinement. Refinement of F^2^ against ALL reflections. The weighted R-factor wR and goodness of fit S are based on F^2^, conventional R-factors R are based on F, with F set to zero for negative F^2^. The threshold expression of F^2^ > 2sigma(F^2^) is used only for calculating R-factors(gt) etc. and is not relevant to the choice of reflections for refinement. R-factors based on F^2^ are statistically about twice as large as those based on F, and R- factors based on ALL data will be even larger.

### Fractional atomic coordinates and isotropic or equivalent isotropic displacement parameters (Å^2^)

**Table:**

	*x*	*y*	*z*	*U*_iso_*/*U*_eq_	
Cl1	0.00707 (4)	1.20793 (2)	1.108564 (19)	0.03204 (9)	
O1	0.79039 (11)	0.47315 (7)	0.97069 (5)	0.0284 (2)	
O2	0.71434 (12)	0.49126 (7)	1.08701 (6)	0.0345 (2)	
N1	0.39801 (11)	0.80622 (7)	1.05570 (5)	0.02072 (19)	
N2	0.39541 (11)	0.84567 (7)	0.93472 (5)	0.01967 (19)	
N3	0.31059 (13)	1.05497 (9)	0.76026 (6)	0.0275 (2)	
N4	0.07811 (15)	1.10407 (11)	0.74881 (7)	0.0405 (3)	
C1	0.47996 (13)	0.73886 (9)	1.01809 (6)	0.0201 (2)	
C2	0.55633 (14)	0.65815 (9)	1.04496 (6)	0.0223 (2)	
H2A	0.5563	0.6408	1.0959	0.027*	
C3	0.63268 (14)	0.60382 (9)	0.99427 (7)	0.0228 (2)	
C4	0.63171 (14)	0.62896 (9)	0.91822 (7)	0.0246 (2)	
H4A	0.6859	0.5905	0.8852	0.030*	
C5	0.55404 (14)	0.70792 (9)	0.89077 (6)	0.0237 (2)	
H5A	0.5516	0.7241	0.8396	0.028*	
C6	0.47921 (13)	0.76287 (9)	0.94225 (6)	0.0201 (2)	
C7	0.34981 (13)	0.86860 (9)	1.00442 (6)	0.0194 (2)	
C8	0.25981 (13)	0.95260 (8)	1.02341 (6)	0.0192 (2)	
C9	0.27664 (13)	1.04393 (9)	0.99159 (6)	0.0214 (2)	
H9A	0.3421	1.0521	0.9523	0.026*	
C10	0.19832 (14)	1.12307 (9)	1.01704 (7)	0.0228 (2)	
H10A	0.2098	1.1850	0.9953	0.027*	
C11	0.10321 (14)	1.11010 (9)	1.07454 (7)	0.0235 (2)	
C12	0.08425 (14)	1.01997 (9)	1.10666 (7)	0.0253 (2)	
H12A	0.0183	1.0121	1.1458	0.030*	
C13	0.16240 (14)	0.94177 (9)	1.08108 (6)	0.0229 (2)	
H13A	0.1498	0.8800	1.1029	0.027*	
C14	0.71494 (14)	0.51778 (9)	1.02296 (7)	0.0255 (2)	
C15	0.87442 (16)	0.38867 (10)	0.99498 (8)	0.0303 (3)	
H15A	0.8083	0.3398	1.0160	0.036*	
H15B	0.9503	0.4068	1.0335	0.036*	
C16	0.94627 (18)	0.34885 (11)	0.92785 (9)	0.0379 (3)	
H16A	1.0040	0.2914	0.9419	0.057*	
H16B	1.0116	0.3979	0.9077	0.057*	
H16C	0.8700	0.3314	0.8901	0.057*	
C17	0.35549 (14)	0.89102 (9)	0.86335 (6)	0.0219 (2)	
H17A	0.2697	0.9342	0.8694	0.026*	
H17B	0.3256	0.8402	0.8271	0.026*	
C18	0.48274 (14)	0.94958 (10)	0.83277 (6)	0.0260 (2)	
H18A	0.5095	1.0027	0.8675	0.031*	
H18B	0.5703	0.9074	0.8286	0.031*	
C19	0.43969 (16)	0.99175 (11)	0.75674 (7)	0.0293 (3)	
H19A	0.5238	1.0290	0.7382	0.035*	
H19B	0.4175	0.9385	0.7214	0.035*	
C20	0.17101 (17)	1.03385 (12)	0.73574 (7)	0.0339 (3)	
H20A	0.1435	0.9750	0.7118	0.041*	
C21	0.16200 (19)	1.17397 (13)	0.78406 (9)	0.0414 (4)	
H21A	0.1252	1.2341	0.8007	0.050*	
C22	0.30538 (18)	1.14512 (11)	0.79179 (8)	0.0349 (3)	
H22A	0.3854	1.1802	0.8144	0.042*	
O1W	0.35783 (15)	0.73706 (9)	0.20403 (6)	0.0449 (3)	
O2W	0.78478 (15)	0.35533 (11)	0.20142 (7)	0.0478 (3)	
H1W1	0.354 (3)	0.7653 (18)	0.1566 (14)	0.067 (7)*	
H2W1	0.307 (2)	0.7811 (16)	0.2306 (12)	0.052 (6)*	
H1W2	0.870 (2)	0.3758 (15)	0.2149 (12)	0.045 (5)*	
H2W2	0.757 (3)	0.3993 (16)	0.1690 (14)	0.057 (6)*	

### Atomic displacement parameters (Å^2^)

**Table:**

	*U*^11^	*U*^22^	*U*^33^	*U*^12^	*U*^13^	*U*^23^
Cl1	0.03324 (18)	0.02542 (16)	0.03796 (17)	0.00757 (13)	0.00755 (13)	−0.00212 (12)
O1	0.0276 (5)	0.0222 (4)	0.0355 (5)	0.0066 (4)	0.0011 (4)	0.0004 (4)
O2	0.0391 (6)	0.0306 (5)	0.0336 (5)	0.0089 (4)	0.0012 (4)	0.0078 (4)
N1	0.0233 (5)	0.0215 (5)	0.0175 (4)	0.0019 (4)	0.0022 (3)	0.0011 (3)
N2	0.0222 (5)	0.0213 (4)	0.0156 (4)	0.0017 (4)	0.0010 (3)	0.0011 (3)
N3	0.0310 (6)	0.0312 (6)	0.0202 (4)	−0.0011 (5)	−0.0003 (4)	0.0056 (4)
N4	0.0324 (6)	0.0539 (8)	0.0351 (6)	0.0007 (6)	0.0024 (5)	0.0165 (6)
C1	0.0211 (5)	0.0217 (5)	0.0174 (4)	−0.0001 (4)	0.0020 (4)	0.0005 (4)
C2	0.0239 (6)	0.0225 (5)	0.0204 (5)	0.0009 (4)	0.0013 (4)	0.0020 (4)
C3	0.0232 (6)	0.0201 (5)	0.0250 (5)	0.0017 (4)	0.0003 (4)	0.0001 (4)
C4	0.0264 (6)	0.0249 (6)	0.0227 (5)	0.0037 (5)	0.0025 (4)	−0.0032 (4)
C5	0.0270 (6)	0.0261 (6)	0.0180 (5)	0.0023 (5)	0.0019 (4)	−0.0015 (4)
C6	0.0209 (5)	0.0217 (5)	0.0176 (4)	0.0010 (4)	0.0006 (4)	0.0003 (4)
C7	0.0201 (5)	0.0213 (5)	0.0168 (4)	−0.0007 (4)	0.0017 (4)	0.0002 (4)
C8	0.0199 (5)	0.0205 (5)	0.0173 (4)	0.0003 (4)	0.0000 (4)	−0.0003 (4)
C9	0.0209 (5)	0.0237 (5)	0.0196 (4)	−0.0008 (4)	0.0004 (4)	0.0021 (4)
C10	0.0231 (5)	0.0200 (5)	0.0251 (5)	0.0004 (4)	−0.0009 (4)	0.0026 (4)
C11	0.0217 (5)	0.0232 (5)	0.0255 (5)	0.0028 (4)	0.0000 (4)	−0.0020 (4)
C12	0.0256 (6)	0.0259 (6)	0.0246 (5)	0.0012 (5)	0.0055 (4)	−0.0002 (4)
C13	0.0246 (6)	0.0219 (5)	0.0225 (5)	0.0005 (4)	0.0041 (4)	0.0017 (4)
C14	0.0237 (6)	0.0209 (5)	0.0317 (6)	0.0011 (5)	−0.0005 (4)	0.0008 (4)
C15	0.0276 (6)	0.0217 (6)	0.0413 (7)	0.0049 (5)	−0.0009 (5)	0.0011 (5)
C16	0.0360 (8)	0.0301 (7)	0.0473 (8)	0.0094 (6)	0.0010 (6)	−0.0014 (6)
C17	0.0240 (5)	0.0268 (6)	0.0149 (4)	0.0021 (5)	−0.0005 (4)	0.0023 (4)
C18	0.0244 (6)	0.0324 (6)	0.0212 (5)	0.0008 (5)	0.0021 (4)	0.0051 (5)
C19	0.0338 (7)	0.0342 (7)	0.0203 (5)	0.0022 (5)	0.0054 (5)	0.0052 (5)
C20	0.0344 (7)	0.0438 (8)	0.0232 (5)	−0.0083 (6)	−0.0025 (5)	0.0084 (5)
C21	0.0451 (9)	0.0374 (8)	0.0421 (8)	0.0076 (7)	0.0070 (7)	0.0098 (6)
C22	0.0406 (8)	0.0317 (7)	0.0323 (6)	−0.0015 (6)	0.0010 (6)	0.0033 (5)
O1W	0.0663 (8)	0.0436 (6)	0.0255 (5)	0.0184 (6)	0.0094 (5)	0.0097 (5)
O2W	0.0364 (6)	0.0639 (8)	0.0424 (6)	−0.0085 (6)	−0.0064 (5)	0.0247 (6)

### Geometric parameters (Å, °)

**Table:**

Cl1—C11	1.7358 (13)	C10—C11	1.3880 (17)
O1—C14	1.3387 (16)	C10—H10A	0.9500
O1—C15	1.4529 (16)	C11—C12	1.3892 (18)
O2—C14	1.2131 (16)	C12—C13	1.3831 (17)
N1—C7	1.3261 (15)	C12—H12A	0.9500
N1—C1	1.3868 (15)	C13—H13A	0.9500
N2—C7	1.3773 (14)	C15—C16	1.504 (2)
N2—C6	1.3782 (15)	C15—H15A	0.9900
N2—C17	1.4646 (14)	C15—H15B	0.9900
N3—C20	1.3544 (18)	C16—H16A	0.9800
N3—C22	1.3729 (19)	C16—H16B	0.9800
N3—C19	1.4642 (18)	C16—H16C	0.9800
N4—C20	1.313 (2)	C17—C18	1.5310 (18)
N4—C21	1.370 (2)	C17—H17A	0.9900
C1—C2	1.3906 (17)	C17—H17B	0.9900
C1—C6	1.4081 (15)	C18—C19	1.5268 (17)
C2—C3	1.3906 (17)	C18—H18A	0.9900
C2—H2A	0.9500	C18—H18B	0.9900
C3—C4	1.4154 (17)	C19—H19A	0.9900
C3—C14	1.4864 (17)	C19—H19B	0.9900
C4—C5	1.3805 (17)	C20—H20A	0.9500
C4—H4A	0.9500	C21—C22	1.361 (2)
C5—C6	1.3979 (16)	C21—H21A	0.9500
C5—H5A	0.9500	C22—H22A	0.9500
C7—C8	1.4687 (16)	O1W—H1W1	0.94 (3)
C8—C9	1.3993 (16)	O1W—H2W1	0.91 (2)
C8—C13	1.4004 (16)	O2W—H1W2	0.85 (2)
C9—C10	1.3933 (17)	O2W—H2W2	0.87 (2)
C9—H9A	0.9500		
			
C14—O1—C15	115.80 (10)	C12—C13—H13A	119.6
C7—N1—C1	105.28 (9)	C8—C13—H13A	119.6
C7—N2—C6	106.64 (9)	O2—C14—O1	123.67 (12)
C7—N2—C17	129.20 (10)	O2—C14—C3	123.52 (12)
C6—N2—C17	123.91 (9)	O1—C14—C3	112.80 (11)
C20—N3—C22	106.48 (13)	O1—C15—C16	106.91 (11)
C20—N3—C19	126.43 (13)	O1—C15—H15A	110.3
C22—N3—C19	127.03 (12)	C16—C15—H15A	110.3
C20—N4—C21	104.98 (13)	O1—C15—H15B	110.3
N1—C1—C2	129.64 (10)	C16—C15—H15B	110.3
N1—C1—C6	109.64 (10)	H15A—C15—H15B	108.6
C2—C1—C6	120.72 (10)	C15—C16—H16A	109.5
C1—C2—C3	117.31 (10)	C15—C16—H16B	109.5
C1—C2—H2A	121.3	H16A—C16—H16B	109.5
C3—C2—H2A	121.3	C15—C16—H16C	109.5
C2—C3—C4	121.46 (11)	H16A—C16—H16C	109.5
C2—C3—C14	117.39 (11)	H16B—C16—H16C	109.5
C4—C3—C14	121.16 (11)	N2—C17—C18	112.44 (10)
C5—C4—C3	121.67 (11)	N2—C17—H17A	109.1
C5—C4—H4A	119.2	C18—C17—H17A	109.1
C3—C4—H4A	119.2	N2—C17—H17B	109.1
C4—C5—C6	116.50 (11)	C18—C17—H17B	109.1
C4—C5—H5A	121.7	H17A—C17—H17B	107.8
C6—C5—H5A	121.7	C19—C18—C17	110.99 (10)
N2—C6—C5	131.86 (10)	C19—C18—H18A	109.4
N2—C6—C1	105.81 (10)	C17—C18—H18A	109.4
C5—C6—C1	122.33 (11)	C19—C18—H18B	109.4
N1—C7—N2	112.63 (10)	C17—C18—H18B	109.4
N1—C7—C8	121.49 (10)	H18A—C18—H18B	108.0
N2—C7—C8	125.88 (10)	N3—C19—C18	111.35 (10)
C9—C8—C13	118.98 (11)	N3—C19—H19A	109.4
C9—C8—C7	123.24 (10)	C18—C19—H19A	109.4
C13—C8—C7	117.52 (10)	N3—C19—H19B	109.4
C10—C9—C8	120.61 (11)	C18—C19—H19B	109.4
C10—C9—H9A	119.7	H19A—C19—H19B	108.0
C8—C9—H9A	119.7	N4—C20—N3	112.24 (14)
C11—C10—C9	119.04 (11)	N4—C20—H20A	123.9
C11—C10—H10A	120.5	N3—C20—H20A	123.9
C9—C10—H10A	120.5	C22—C21—N4	110.43 (15)
C10—C11—C12	121.30 (11)	C22—C21—H21A	124.8
C10—C11—Cl1	120.00 (10)	N4—C21—H21A	124.8
C12—C11—Cl1	118.70 (9)	C21—C22—N3	105.87 (14)
C13—C12—C11	119.30 (11)	C21—C22—H22A	127.1
C13—C12—H12A	120.3	N3—C22—H22A	127.1
C11—C12—H12A	120.3	H1W1—O1W—H2W1	101.6 (19)
C12—C13—C8	120.77 (11)	H1W2—O2W—H2W2	101 (2)
			
C7—N1—C1—C2	179.48 (12)	C7—C8—C9—C10	−173.62 (11)
C7—N1—C1—C6	−0.08 (13)	C8—C9—C10—C11	0.05 (17)
N1—C1—C2—C3	−178.60 (12)	C9—C10—C11—C12	−0.39 (18)
C6—C1—C2—C3	0.91 (18)	C9—C10—C11—Cl1	178.71 (9)
C1—C2—C3—C4	−0.60 (18)	C10—C11—C12—C13	0.39 (19)
C1—C2—C3—C14	179.73 (11)	Cl1—C11—C12—C13	−178.73 (10)
C2—C3—C4—C5	−0.6 (2)	C11—C12—C13—C8	−0.04 (19)
C14—C3—C4—C5	179.08 (12)	C9—C8—C13—C12	−0.30 (18)
C3—C4—C5—C6	1.37 (19)	C7—C8—C13—C12	173.96 (11)
C7—N2—C6—C5	−179.35 (13)	C15—O1—C14—O2	−0.03 (19)
C17—N2—C6—C5	5.9 (2)	C15—O1—C14—C3	179.57 (10)
C7—N2—C6—C1	0.18 (13)	C2—C3—C14—O2	2.6 (2)
C17—N2—C6—C1	−174.58 (11)	C4—C3—C14—O2	−177.04 (13)
C4—C5—C6—N2	178.41 (12)	C2—C3—C14—O1	−176.97 (11)
C4—C5—C6—C1	−1.06 (19)	C4—C3—C14—O1	3.36 (17)
N1—C1—C6—N2	−0.07 (13)	C14—O1—C15—C16	178.52 (12)
C2—C1—C6—N2	−179.67 (11)	C7—N2—C17—C18	108.32 (14)
N1—C1—C6—C5	179.52 (11)	C6—N2—C17—C18	−78.16 (14)
C2—C1—C6—C5	−0.08 (19)	N2—C17—C18—C19	177.29 (10)
C1—N1—C7—N2	0.20 (14)	C20—N3—C19—C18	−105.09 (14)
C1—N1—C7—C8	−178.91 (10)	C22—N3—C19—C18	71.78 (17)
C6—N2—C7—N1	−0.24 (14)	C17—C18—C19—N3	59.23 (15)
C17—N2—C7—N1	174.15 (11)	C21—N4—C20—N3	−0.39 (16)
C6—N2—C7—C8	178.82 (11)	C22—N3—C20—N4	0.51 (15)
C17—N2—C7—C8	−6.79 (19)	C19—N3—C20—N4	177.91 (11)
N1—C7—C8—C9	140.42 (12)	C20—N4—C21—C22	0.12 (17)
N2—C7—C8—C9	−38.57 (18)	N4—C21—C22—N3	0.19 (17)
N1—C7—C8—C13	−33.57 (16)	C20—N3—C22—C21	−0.41 (15)
N2—C7—C8—C13	147.44 (12)	C19—N3—C22—C21	−177.78 (12)
C13—C8—C9—C10	0.29 (17)		

### Hydrogen-bond geometry (Å, °)

**Table:**

Cg1 is the centroid of the C1–C6 phenyl ring.

**Table:**

*D*—H···*A*	*D*—H	H···*A*	*D*···*A*	*D*—H···*A*
O1W—H1W1···N1^i^	0.94 (3)	1.96 (3)	2.8802 (14)	164 (2)
O1W—H2W1···O2W^ii^	0.91 (2)	1.83 (2)	2.7284 (18)	169 (2)
O2W—H1W2···N4^iii^	0.849 (19)	1.978 (19)	2.8147 (19)	169 (2)
O2W—H2W2···O2^i^	0.87 (2)	1.98 (2)	2.8460 (17)	172 (2)
C17—H17B···O1W^iv^	0.99	2.49	3.3785 (16)	149
C19—H19B···O1W^iv^	0.99	2.51	3.3799 (19)	147
C10—H10A···Cg1^v^	0.95	2.86	3.4875 (14)	125

Symmetry codes: (i) *x*, *y*, *z*−1; (ii) −*x*+1, *y*+1/2, −*z*+1/2; (iii) *x*+1, −*y*+3/2, *z*−1/2; (iv) *x*, −*y*+3/2, *z*+1/2; (v) −*x*+1, −*y*+2, −*z*+2.
